# Methylation biomarkers can distinguish pleural mesothelioma from healthy pleura and other pleural pathologies

**DOI:** 10.1002/1878-0261.70159

**Published:** 2025-11-14

**Authors:** Janah Vandenhoeck, Nele De Meulenaere, Thomas Vanpoucke, Joe Ibrahim, Dieter Peeters, Suresh Krishan Yogeswaran, Wen Wen, Paul Van Schil, Jeroen M. H. Hendriks, Jo Raskin, Jan van Meerbeeck, Guy Van Camp, Ken Op de Beeck

**Affiliations:** ^1^ Center of Medical Genetics University of Antwerp and Antwerp University Hospital Edegem Belgium; ^2^ Center for Oncological Research Antwerp (CORE) University of Antwerp and Antwerp University Hospital Wilrijk Belgium; ^3^ Department of Pathology Antwerp University Hospital and University of Antwerp Edegem Belgium; ^4^ Department of Thoracic and Vascular Surgery Antwerp University Hospital Edegem Belgium; ^5^ Antwerp Surgical Training, Anatomy and Research Center (ASTARC) University of Antwerp Wilrijk Belgium; ^6^ Department of Thoracic Oncology Antwerp University Hospital and University of Antwerp Edegem Belgium

**Keywords:** cancer biomarkers, DNA methylation, pleural mesothelioma

## Abstract

Pleural mesothelioma (PM) is a rare and aggressive cancer that often requires multiple diagnostic procedures before a definitive diagnosis can be made. To improve diagnostic accuracy, we developed a DNA methylation‐based biomarker assay capable of distinguishing PM from healthy pleura and other pleural pathologies. Using Infinium EPIC array data, we identified 744 hypermethylated CpG sites in PM as candidate biomarkers. These were validated *in silico* using external datasets, yielding a high mean AUC of 0.935. Clinical validation was performed using IMPRESS, a novel bisulfite‐free methylation detection technique that enables simultaneous analysis of thousands of CpG sites. A two‐step classifier approach was applied: the first model differentiated tumoral from nontumoral pleura with 89.2% sensitivity and 93.5% specificity, while the second model distinguished PM from pleural metastases with 85.2% sensitivity and 100% specificity. These results demonstrate that our methylation‐based biomarker panel offers a highly accurate and minimally invasive tool for differentiating PM from other pleural conditions, potentially streamlining the diagnostic process and improving clinical decision‐making.

## Graphical abstract

We developed and validated a DNA methylation–based biomarker panel to distinguish pleural mesothelioma from other pleural conditions. Using the IMPRESS technology, we translated this panel into a clinically applicable assay. The resulting two classifier models demonstrated excellent performance, achieving high AUC values and strong diagnostic accuracy.

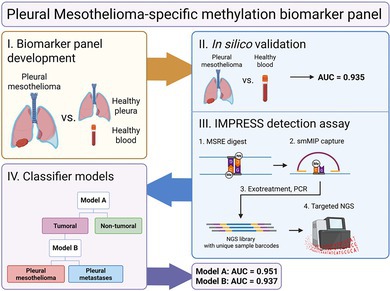

**Abbreviations**
CT: computed tomography
cvAUC: cross‐validated area under the curve
DMC: differentially methylated CpG site
IMPRESS: improved methylation profiling using restriction enzymes and smMIP sequencing
LDA: linear discriminant analysis
MDS: multidimensional scaling
MSRE: methylation‐sensitive restriction enzyme
PM: pleural mesothelioma
ROC: receiver operating characteristic
smMIP: single molecule molecular inversion probe
SVD: singular value decomposition

## 1 Introduction

Pleural mesothelioma (PM) is a rare and aggressive tumor originating from the mesothelial cells of the pleura [1]. Histologically, PM can be categorized into three main subtypes: epithelioid (50–60% of all cases, with the best prognosis), sarcomatoid (10–20% of all cases, with the worst prognosis), and biphasic (30% of all cases, combining characteristics of both) [2]. Exposure to asbestos accounts for over 80% of PM cases, with symptoms typically appearing after a latency period of 30–50 years [3]. In 2020, an estimated 30 870 deaths worldwide were attributed to PM, with the highest numbers reported in Northern Europe [4]. However, due to underreporting and frequent misdiagnoses, accurately assessing the global burden remains challenging. Although the incidence rate has begun to decline in some countries with asbestos bans, only 70 countries worldwide have prohibited its use [4, 5]. Consequently, PM is expected to remain a significant global health issue in the coming years.

Due to the challenges and frequent delays in diagnosis, tumors are often detected at an advanced stage, resulting in a poor prognosis. Treatment is mostly palliative in intention, with a combination of immune checkpoint inhibitors ipilimumab and nivolumab, as the first‐line standard of care in most countries [6, 7]. With this dual immunotherapy, a median life expectancy of 18 months was reported, compared to 9–12 months in untreated patients [7]. Platinum and pemetrexed chemotherapy is administered as a second‐line therapy in fit relapsing patients. Surgery in rare early‐stage cases remains controversial [8].

Diagnosing PM is challenging due to several factors. Its silent clinical progression and/or nonspecific symptoms, such as shortness of breath, chest pain, and cough, often lead to diagnostic delays [7, 9]. Moreover, PM may be difficult to distinguish from other benign or malignant pleural diseases, including chronic pleuritis and pleural metastases [3, 10]. The current diagnostic approach typically involves a computed tomography (CT) scan, followed by histopathological examination of a tissue biopsy [11, 12]. However, detecting PM tumors on CT scans can be challenging, as visualization often requires tumors to reach a certain size and their diffuse, nonspherical shape further complicates detection [3, 13]. Additionally, CT scans may be unreliable in differentiating PM from other pleural malignancies [14], making biopsy‐based histopathological analysis essential for accurate diagnosis [3]. Tissue samples are obtained through thoracoscopy or image‐guided needle biopsy.

Pathological analysis serves two purposes [11]. First, the cellular origin is determined using a panel of immunohistochemical markers [10, 11]. The differential diagnosis of epithelioid mesotheliomas from other pleural tumors, especially pleural metastases derived from carcinomas, can be challenging and typically requires multiple mesothelial markers (e.g., calretinin, WT1, D2‐40, CK5/6) and epithelial markers (e.g., claudin‐4, BerEP4, MOC‐31, B72.3, CEA) [11, 15]. Pancytokeratins are also helpful, as sarcomatoid mesotheliomas are generally at least focally positive for pancytokeratin, while a negative result may suggest a nonmesothelial tumor [15]. However, none of these markers is 100% sensitive or specific for mesothelial lineage. For instance, only about 30% of sarcomatoid mesotheliomas express calretinin, although D2‐40 is more frequently positive [15]. Other mesothelial markers, such as CK5/6 and WT1, are also frequently negative in sarcomatoid cases [15].

Second, malignancy must be assessed by cellular morphology or molecular testing. Morphological analysis focuses on invasive growth patterns, especially into underlying adipose tissue. This is essential to differentiate PM from reactive mesothelial changes in chronic pleuritis [12]. Additionally, molecular markers such as loss of *BAP1* expression, homozygous deletion of *CDKN2A* (detected by FISH), or loss of MTAP expression (indicating *CDKN2A* deletion) can help distinguish PM from benign proliferations [11, 15]. Still, these markers are not entirely specific in differentiating PM from other pleural malignancies. Accurate PM diagnosis therefore often requires a combination of immunohistochemical markers and molecular analyses. However, these depend on large, high‐quality tissue samples, which are often limited in tissue biopsies. Furthermore, interpathologist diagnostic variability is common [16]. These limitations highlight the need for a comprehensive and reliable assay that complements current histology while requiring minimal tissue.

Another key challenge in PM diagnosis is its molecular heterogeneity, both within individual tumors and between patients [17]. Molecular analyses show variability across distinct tumor regions and over treatment trajectories [18]. PM has a heterogeneous genetic landscape, with a relatively low somatic mutational burden, mainly involving tumor suppressor gene inactivation, such as *BAP1* (45.1% prevalence), *NF2* (31.3%), *CDKN2A* (42.2%), *CDKN2B* (36.0%), *MTAP* (27.3%), and *TP53* (17.3%) [8, 19]. Copy number alterations are also observed, with large chromosomal losses on Chromosomes 1, 3, 4, 6, 9, 13, and 22 in at least 25% of patients, and gains on Chromosomes 1, 5, 7, and 17 in at least 15% [20].

Given PM's low mutation and copy number burden, increasing attention is turning toward the epigenome, which holds great potential as a biomarker source. The epigenome includes histone modifications (e.g., acetylation, phosphorylation, and methylation) and DNA alterations such as methylation, without altering the DNA sequence [21]. DNA methylation occurs when a methyl group is added to the fifth carbon of cytosine in a CpG dinucleotide, producing 5‐methylcytosine [22]. Compared to mutations, DNA methylation is more stable and shows less intratumor heterogeneity in PM, making it a promising biomarker target [21].

In various tumors, global epigenetic reprogramming occurs early during tumorigenesis, suggesting that epigenetic changes may serve as useful early detection biomarkers [23]. Generally, global hypomethylation leads to genomic instability, while hypermethylation in specific CpG islands may result in gene silencing or inactivation [24, 25]. In PM, DNA methylation of individual genes has been studied previously [26], but research has recently begun to explore genome‐wide methylation patterns [10, 27]. Our group recently described a PM‐specific genome‐wide methylation signature by comparing PM samples to healthy pleura and other lung‐related diseases [28]. This study assesses the biomarker potential of that signature by selecting and validating differentially methylated CpG sites between PM, healthy pleura, and blood samples.

To this end, we employed IMPRESS (Improved Methylation Profiling using Restriction Enzymes and smMIP Sequencing) [29], a novel, low‐cost detection method that enables simultaneous methylation analysis in thousands of targeted regions. IMPRESS combines four methylation‐sensitive restriction enzymes (MSREs) with single‐molecule Molecular Inversion Probes (smMIPs), resulting in a targeted NGS panel that significantly reduces costs compared to genome‐wide sequencing. Moreover, unlike bisulfite‐based methods, no DNA is degraded by IMPRESS, making it especially suited for low‐input applications such as liquid biopsies [30].

The aim of this study was to establish a methylation‐based biomarker panel capable of differentiating PM from healthy pleura and diagnostic confounders using a single, easy and comprehensive assay. We also sought to validate this biomarker panel both *in silico* and on clinical samples using IMPRESS. By selecting the best‐performing smMIPs to develop two classification models, we created an assay for implementation in clinical diagnostic settings to complement existing histopathological analyses.

## 2 Materials and methods

### 2.1 Sample collection and processing

Tissue samples from patients with pleural diseases were collected at the Antwerp University Hospital biobank (UZA, Belgium) between January 2012 and November 2021. These patients suffered from pleural mesothelioma (*n* = 28), pleural metastases of other malignancies (*n* = 10), or chronic pleuritis (*n* = 8) (Table S1). Only samples from treatment‐naïve PM patients were used. Additionally, 47 healthy pleural tissue samples were obtained from patients undergoing thoracic surgery for reasons other than PM (Table S1). These samples were collected at UZA between September 2021 and November 2023. Nineteen of these were used for biomarker panel development and 28 for IMPRESS validation (Table 1, Table S2). All tissue samples were fresh‐frozen and stored at −80 °C until further use. Diagnosis and tumor cell percentage (TCP) were confirmed by a pathologist (D.P.) through histological examination. DNA was extracted from ten to fifteen 10‐μm sections using the QIAamp DNA Micro Kit (Qiagen, Hilden, Germany) according to the manufacturer's protocol, and stored at −20 °C until further use. Furthermore, 11 whole blood samples from healthy volunteers were collected and genomic DNA (gDNA) was extracted via a salting‐out process and stored at 4 °C until further use.

**Table 1:** Overview of the samples used in the different parts of this study. More information about the online available datasets is given in Table S2.

**Discovery: CpG site selection**	
134 Pleural mesothelioma	Online available data [66]
22 Healthy pleura	Online available data (*n* = 3) [66] In‐house sample collection (*n* = 19)
143 Healthy blood	Online available data [67, 68, 69]
***In silico* validation: external datasets**	
121 Pleural mesothelioma	Online available data [10, 27, 70, 71, 72, 73, 74, 75, 76]
169 Healthy blood	
**Wet lab validation: IMPRESS experiments**	
28 Pleural mesothelioma	In‐house sample collection
10 Pleural metastases	
8 Chronic pleuritis	
28 Healthy pleura	
11 Healthy blood	

### 2.2 Development of a pleural mesothelioma‐specific methylation biomarker panel

Candidate methylation biomarkers were identified using both publicly available Infinium Human Methylation 850K EPIC BeadChip array data (v1.0) and in‐house generated array data (v1.0 and v2.0) (Table 1), and processed as described by Ibrahim et al. [24]. Data from the v1.0 arrays were normalized separately from the v2.0 arrays, and subsequently, probes common to all datasets were retained for downstream analysis. Possible batch effects were assessed using Singular Value Decomposition (SVD) analysis, which showed that the sample group was the main source of variation, so batch effect correction was omitted to preserve the biological signal. Differentially methylated CpG sites (DMCs) were identified as candidate biomarkers using the champ package in r (version 4.0.2), by comparing PM to both healthy pleura and healthy blood. Specifically, only significantly DMCs (adjusted *P*‐value ≤ 0.05) that are hypermethylated in PM and located within recognition sites of the four MSREs used in IMPRESS were selected. Moreover, for the healthy controls, we applied an average methylation cutoff of a maximum of 0.3 and a standard deviation cutoff of a maximum of 0.1. We selected the top 1000 hypermethylated DMCs in PM, ranked by methylation difference between PM and both control groups. After selecting the DMCs for the final models, possible batch effects were tested for each DMC individually using linear mixed models and ANOVA. After FDR correction, no significant batch effects were found.

Using the MIPGEN software, smMIPs were designed for both DNA strands (double‐tiled) for each selected target site [31]. Each smMIP includes a 30 nt backbone, 5 nt single‐molecule tags on both ends, and two binding arms of approximately 20 nt, flanking a 50 nt insert.

### 2.3 *In silico* validation of the pleural mesothelioma‐specific methylation biomarker panel

The biomarker panel was validated *in silico* on 121 PM and 169 healthy blood samples, from public Infinium Human Methylation 850K EPIC array data (Table 1). For each target, a linear discriminant analysis (LDA) model was trained on the discovery cohort with the mass package in r (version 4.0.2) and tested on the validation cohort, with AUC values calculated using the rocr package.

### 2.4 Validation of the pleural mesothelioma‐specific methylation biomarker panel on clinical samples

#### 2.4.1 IMPRESS assay

The DNA methylation analysis was executed using the IMPRESS assay, which combines methylation‐sensitive restriction enzyme (MSRE) digestion with single‐molecule Molecular Inversion Probe (smMIP) sequencing [29]. A patent application is pending for this technology. Unmethylated lambda phage DNA was spiked into each sample to serve as an internal control for the MSRE digest. Lambda smMIPs targeting MSRE recognition sites (*n* = 12) and reference sites without CpG sites (*n* = 10) in lambda DNA were used to calculate the percentage of undigested fragments. Human reference smMIPs, targeting regions without CpG sites (*n* = 300) or without MSRE recognition sites (*n* = 300), were used to normalize the read counts and correct for the amount of input DNA. All human reference and lambda smMIPs were identical to those described by Vandenhoeck et al. [29]. The analysis of the NGS output (High Output Kit, Nextseq 550, Illumina, San Diego, CA, USA) was performed using an in‐house developed bioinformatic Snakemake pipeline, as described by Vandenhoeck et al. [29]. In short, sequencing reads were deconvoluted per sample and mapped to the reference genome (hg19) using the *bwamem* algorithm. After duplicate removal (*Picard MarkDuplicates*) and quality filtering, reads per smMIP location were counted for each sample, obtaining a dataset with counts for all smMIPs for each sample.

#### 2.4.2 Classifier model

The classifier models were developed as described by Vandenhoeck et al. [29]. For each model, the most efficient and discriminating smMIPs were selected. Initially, the least efficient smMIPs were removed using a cutoff of 1000 cumulative counts across all undigested samples to ensure robust biomarkers. For each remaining smMIP, LDA was performed using the mass package in R (version 4.0.2) [32]. An LDA model was constructed using fivefold cross‐validation. This was carried out with a randomization restriction to proportionally represent the tissue types across the fivefolds. The mean cross‐validated area under the curve (cvAUC) was calculated for each smMIP using the rocr package [33]. smMIP models with a cvAUC below 0.8 were excluded from the final model. The prediction cutoff for each smMIP model was determined based on the highest accuracy. In cases involving double‐tiled smMIPs, only the best‐performing one was retained. For each model, a boxplot was generated based on the sum of the normalized counts for the selected smMIPs. Statistical significance was assessed using Welch's *t*‐test (unequal variance) in r (version 4.3.2). Additionally, a multidimensional scaling (MDS) plot was created for each model using the selected smMIPs. The remaining single smMIP models were then combined into a final comprehensive model. This comprehensive model was assessed using a receiver operating characteristic (ROC) curve, after which the final prediction cutoff was determined to achieve the highest overall accuracy for this final classifier model.

### 2.5 Ethics approval and consent to participate

This study was conducted in accordance with Good Clinical Practice guidelines and the Declaration of Helsinki. Fresh‐frozen tissue samples used in this study were previously collected in the Biobank of the Antwerp University Hospital and retrospectively used in this study. According to Article 20 of the Belgian Law on the procurement and use of human corporal material intended for human application or scientific research of 19 December 2008, patients provide consent for the use of their bodily material in research when consenting to an invasive procedure. As such, for all patients suffering from PM, metastases in the pleura of other malignancies or chronic pleuritis, no additional consent was needed for using retrospective samples. For prospectively collected healthy pleural tissue samples, written informed consent was given by each subject. The healthy pleural tissue was obtained from the macroscopically intact parietal pleura at a distance from any gross intrathoracic disease. The study protocol and any modifications thereof were approved by the UZA ethical committee (Reference number 16/23/248 and EDGE number 002046).

## 3 Results

### 3.1 Development of a pleural mesothelioma‐specific methylation biomarker panel

EPIC methylation data from 134 PM samples, three healthy pleura samples, and 143 healthy blood samples were obtained from public datasets. Additionally, we generated in‐house 850K EPIC array data of 19 healthy pleura samples from the Antwerp University Hospital (UZA). These data were used to identify potential biomarkers (Table 1). The methylation profiles of PM were compared to those of both healthy pleura and blood to identify significantly differentially methylated CpG sites (DMCs), with overlapping DMCs retained for further analysis. Only DMCs located within recognition sites of the four MSREs used in IMPRESS were selected. For the healthy controls, we applied an average methylation cutoff of a maximum of 0.3 and a standard deviation cutoff of a maximum of 0.1. We selected the top 1000 hypermethylated DMCs in PM, ranked by methylation difference between PM and both control groups. smMIPs were designed for each target using MIPGEN [31], and after filtering out 256 unsuitable targets (impossible probe design or SNP/repeat captured), 1229 smMIPs covering 744 CpG sites were retained, including 270 single‐, 463 double‐ and 11 triple‐tiled sites (Table S3).

### 3.2 *In silico* validation of the biomarker panel using external methylation datasets

Following biomarker selection and smMIP design, newly available EPIC methylation array data from 121 PM samples and 169 healthy blood samples enabled an *in silico* validation. To avoid bias, the number of healthy blood samples was limited to match the PM group. Data on healthy pleura samples were still unavailable. The analysis focused on 614 of the 744 DMCs with qualitative data across all validation datasets. Trained on the discovery cohort and tested in the validation cohort, these DMCs showed strong discriminatory power, with a mean AUC of 0.935 (range: 0.684–1; median: 0.951). These results confirm the biomarker potential of the selected DMCs.

### 3.3 Pleural mesothelioma detection assay

#### 3.3.1 Data processing

To validate the biomarker panel in the laboratory, we analyzed 28 PM tissue, 28 healthy pleura, 11 healthy blood, and 18 diagnostic confounder samples (10 pleural metastases and eight chronic pleuritis) using the IMPRESS technique. Each sample was targeted by 1851 smMIPs (1229 PM targets, 600 human reference, and 22 lambda controls), and read counts were obtained for each smMIP. Unmethylated lambda phage DNA served as an internal control for MSRE digest efficiency. Samples with > 5% undigested fragments were excluded, which applied to six out of 88 samples (1 PM and 5 healthy pleura samples). All remaining samples showed a mean percentage of 1.7% undigested fragments.

PM smMIP read counts were normalized using human reference smMIPs to account for input DNA, by dividing each PM smMIP count by the total reference smMIP count per sample. Higher normalized counts indicate methylation (PM samples), while lower counts reflect nonmethylated samples (healthy pleura and blood). When analyzing the sample types used in the discovery cohort (i.e., PM, healthy pleura, and healthy blood), PM samples showed significantly higher normalized counts (0.69 ± 0.32) than healthy pleura (0.24 ± 0.07) and healthy blood (0.18 ± 0.03) (Fig. S1).

#### 3.3.2 Classifier models

**Fig. 1:**
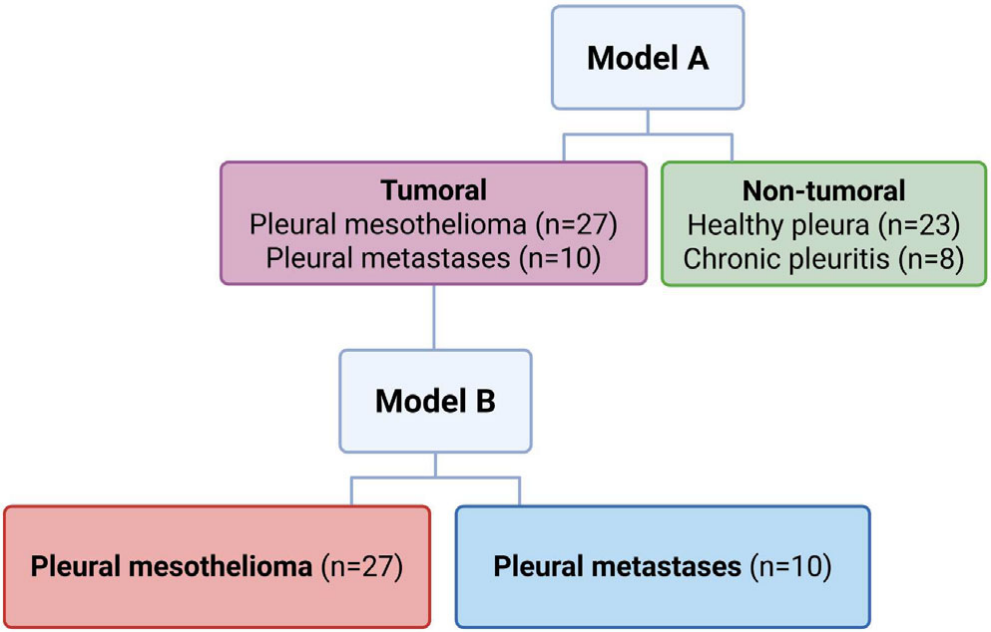
Overview of the stepwise classifier models with sample types and numbers included. If samples are classified as tumoral by Model A, a further distinction between pleural mesothelioma (PM) and pleural metastases can be made by Model B.

Classifier models were developed to distinguish PM from key diagnostic confounders using a clinically relevant stepwise approach. Model A separates tumoral (PM and pleural metastases) and nontumoral (healthy pleura and chronic pleuritis) conditions, while Model B distinguishes PM from pleural metastases (Fig. 1).

**Fig. 2:**
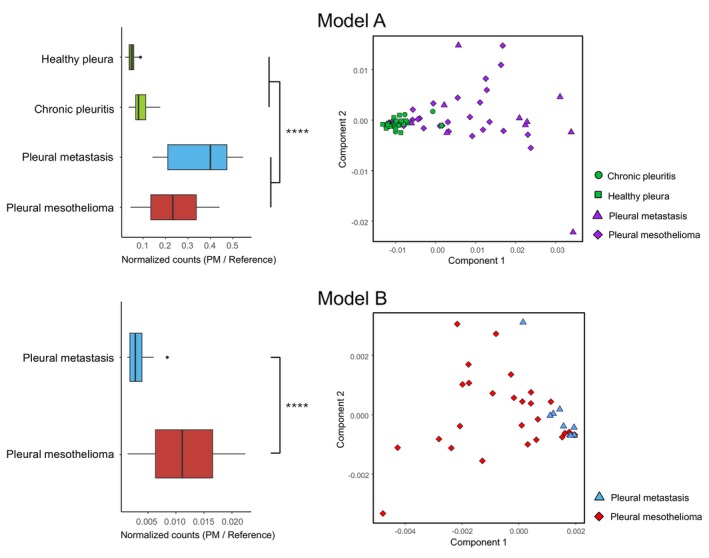
Boxplots and MDS plots for classifier Model A and Model B. The boxplots show the sample distribution of the sum of the normalized counts for the subselection of PM smMIPs in each model. In Model A, based on a subselection of 310 smMIPs, 27 pleural mesothelioma samples and 10 pleural metastases (red and blue, or purple) are differentiated from 23 healthy pleura and 8 chronic pleuritis samples (green). In Model B, based on a subselection of 21 smMIPs, 27 pleural mesothelioma samples (red) are differentiated from 10 pleural metastasis samples (blue). ****P‐value ≤ 0.0001. The unequal variance (Welch) *t*‐test was performed. MDS, multidimensional scaling; PM, pleural mesothelioma; smMIPs, single molecule molecular inversion probes.

For both classifier models, the most efficient and discriminating smMIPs were selected. The distribution of the cvAUC values per smMIP for each model is shown in Fig. S2. For all double‐tiled targets (i.e., CpG sites targeted by multiple smMIPs), only the best‐performing smMIP per target was selected, resulting in 310 smMIPs for Model A and 21 smMIPs for Model B (Tables S4 and S5). Sample distributions, based on the sum of the normalized counts of the smMIP subselection, are shown in Fig. 2, with both models showing strong statistical significance (Model A: *P* = 5.4 × 10^−11^; Model B: *P* = 3.3 × 10^−6^). Additionally, a clustering analysis was performed for each model using multidimensional scaling (MDS) with the respective smMIP selections (Fig. 2).

**Fig. 3:**
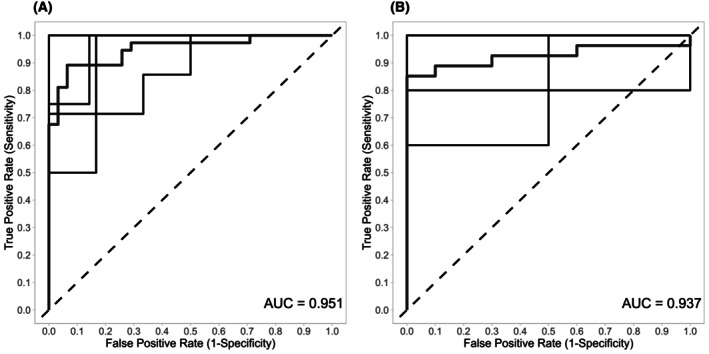
ROC curves for classifier Model A (A) and Model B (B). These graphs display the trade‐off between sensitivity (true positive rate) and 1 − specificity (false positive rate) across different cutoff values of a classification model. The black lines in both ROC plots represent the five groups of the cross‐validation; the red line represents the mean cross‐validated ROC curve. Model A enables a distinction between tumoral (*n* = 37) and non‐tumoral samples (*n* = 31); Model B differentiates pleural mesothelioma (*n* = 27) from pleural metastases (*n* = 10). AUC, area under the curve; ROC, receiver operating characteristic.

The remaining single smMIP models were combined into a final comprehensive model, with ROC curves shown in Fig. 3A,B. The optimal prediction cutoffs, determining the number of single smMIP models required to agree on the classification, were chosen for the highest overall accuracy, being 91 for Model A and 11 for Model B. Model performance metrics, including cvAUC value, sensitivity, and specificity, are detailed in Table 2.

**Table 2:** Metrics of both classifier models.

	Model A	Model B
Selected smMIPs	310	21
cvAUC	0.951	0.937
True positives	33	23
True negatives	29	10
False positives	2	0
False negatives	4	4
Sensitivity	0.892	0.852
Specificity	0.935	1.00
Accuracy	0.912	0.892
Balanced accuracy	0.914	0.926
Positive predictive value	0.943	1.00
Negative predictive value	0.879	0.714

Model A includes 310 target sites, located in intergenic regions and within 174 genes. Among these genes, 22 contain multiple targets: *AXL1, CD8A, DLX1, DLX6AS, EBF3, EMX1, FLJ41350, FOXD3, GCM2, NPY, NXPH1, PAX6, PENK, SEZ6L2, STAP2, TCTEX1D1, TLX3, ZIC4* (each with two targets), *C2orf55, CLDN10*, and *PRDM14* (three targets) and *ZIC1* (five targets). Full details of all 310 target sites are provided in Table S6. Model B comprises 21 target sites within 18 genes and in intergenic regions. The 18 genes in Model B are *ACCN4, ANGPTL6, ATOH8, CDC42EP5, CDH4, CFD, FOXN3, GRHL2, HOXA9, KISS1R, KRT86, NCKAP5, PKP1, POMC, TCF7L1, THRB, TNFRSF10C*, and *TRIL* (Table S7).

## 4 Discussion

To diagnose PM, many tissue markers have been identified and are clinically used [11, 15]. However, none alone provides sufficient sensitivity or specificity to reliably distinguish PM from all diagnostic confounders [11, 15]. As a result, multiple markers are usually required to reach a conclusion, making the process costly, time‐consuming, and dependent on sufficient tissue for immunohistochemical staining and molecular testing.

Methylation biomarkers hold strong potential for cancer diagnostics due to their stability during storage and accessibility in liquid biopsies. The Epi proLung test, detecting aberrant methylation of *SHOX2* and *PTGER4* in lung cancer blood samples, exemplifies this potential [34]. By targeting a single gene, it remains cost‐effective, offering a practical diagnostic tool for early cancer detection. However, its success depends on one or a few genes with consistently deregulated methylation in lung cancer, which have not yet been found in PM. This complicates the design of single‐gene assays. Notwithstanding, methylation panels or multigene strategies could address intertumor heterogeneity. DNA methylation‐based classification has also revolutionized central nervous system tumor diagnostics [35]. The Heidelberg classification system, for example, leverages methylation profiles for highly accurate tumor subtyping, even in ambiguous cases. This approach enhances diagnostic precision, guiding personalized treatment.

Epigenetic studies in PM have identified numerous CpG sites and islands with aberrant methylation patterns [10, 27, 36, 37, 38]. Bertero et al. [27] used unsupervised clustering and t‐SNE analysis to define a methylation profile distinguishing PM from diagnostic confounders. Similarly, Jurmeister et al. [10] developed a PM‐specific methylation signature that distinguishes PM from chronic pleuritis, pleural carcinosis, and pleomorphic lung carcinomas using random forest and support vector machine learning, with accuracies of 89.5% and 97.8%, respectively, on *in silico* data. These studies highlight the potential of methylation profiling to distinguish PM from other conditions. Additionally, studies by Allione et al. and Guarrera et al. identified DMCs in whole‐blood DNA as potential PM biomarkers, both achieving AUC values of 89% [36, 37]. Yet, translation of these methylation biomarkers into a clinically relevant assay has been lacking. Our study addresses this gap by developing a methylation biomarker panel, designing a detection assay, and validating its diagnostic utility in an external set of clinical samples. We employ IMPRESS, a sensitive, cost‐efficient, multi‐target, bisulfite‐free detection technique suitable for clinical use. This represents a key step toward the practical adoption of methylation biomarkers in PM diagnosis.

We developed a methylation‐based biomarker panel consisting of 744 hypermethylated CpG sites in PM, by comparing methylation profiles of PM with healthy pleura and blood. The unique collection of healthy pleural tissue samples enabled a direct comparison with healthy pleural tissue, avoiding reliance on normal adjacent samples. Incorporating healthy blood data for the biomarker selection ensured potential for future liquid biopsy applications. We initially validated the biomarker panel *in silico* using external datasets, achieving a mean AUC of 0.935, indicating strong performance. We then tested the panel in the wet lab using IMPRESS on 28 PM tissue, 28 healthy pleura, 11 healthy blood, and 18 diagnostic confounder samples.

Using IMPRESS assay data, we developed a two‐step classifier model to distinguish PM from its key diagnostic confounders, based on specific smMIP selections targeting CpG sites with different methylation levels (Fig. 1). The first model differentiates tumoral (PM and pleural metastases) from nontumoral (healthy pleura and chronic pleuritis) conditions, using 310 smMIPs, achieving 89.2% sensitivity and 93.5% specificity. The second model separates PM from pleural metastases, using 21 smMIPs. Despite this limited smMIP number, this model achieves an impressive specificity of 100% and sensitivity of 85.2%. Since the initial 744 CpG sites were selected without including other pleural pathologies, some DMCs may not be exclusively PM‐specific and could have had an overlap with other conditions, such as pleural metastases. However, the 21 smMIPs used in the second model were specifically chosen for their ability to distinguish PM from pleural metastases, ensuring their accuracy in differentiation.

Biomarker selection was independently tailored for each classification, resulting in distinct, nonoverlapping CpG sets. Combined, the two‐step approach effectively differentiates PM from both nontumoral tissue and pleural metastases.

Model A targets 310 CpG sites across intergenic regions and within 174 unique genes. Twenty‐two of these genes contain multiple selected CpG sites. Three genes (*FOXD3*, *PENK*, and *ZIC1*) have been described to play a role in PM, while two (*PAX6* and *TCTEX1D1*) have been associated with asbestos exposure. The remaining genes have been described in other cancer types. FOXD3, a transcriptional regulator of pluripotent stem cells, is reported to be downregulated in PM and other cancers [39, 40]. *PENK* has been reported to undergo methylation‐associated silencing in PM [41] and shows aberrant methylation in cancers such as colorectal and bladder [42, 43]. The tumor suppressor gene *ZIC1* is epigenetically silenced in several cancers, including PM [44, 45], where its promoter methylation lowers expression, promoting tumor growth [44]. ZIC1 also suppresses oncogenic miRNAs *miR‐23a* and *miR‐27a*, whose overexpression is linked to poor survival [44]. Regarding asbestos‐associated genes, *PAX6* expression has been linked to asbestos exposure [46] and it shows aberrant methylation in squamous cell carcinomas [47]. *TCTEX1D1*, although not yet linked to PM, has a SNP associated with asbestos‐related lung cancer [48] and shows methylation changes in lung and clear cell renal cell cancer onsets [49, 50].

All 18 genes with CpG sites in Model B have been previously described in cancer. Six of these (*THRB*, *CDH4*, *KISS1R*, *HOXA9*, *POMC*, and *FOXN3*) have been reported in PM, while *CFD* was found in a protein interaction network in peritoneal mesothelioma [51]. In PM, the tumor suppressor gene *THRB* shows both SNPs and altered expression [52]. CDH4 expression is positively associated with epithelial PM [53]. Both genes also display methylation changes in cancers such as colorectal, gastric, and breast [54, 55, 56, 57]. *HOXA9* hypermethylation serves as both a biomarker for detection and recurrence in multiple cancers [58, 59, 60]. Its strong expression in PM suggests it as a therapeutic target [61], and it is included in a PM detection panel for malignant pleural effusions [62]. POMC expression has been reported in PM cell lines involved in growth‐promoting pathways [63]. FOXN3 downregulation, linked to TUSC2 loss, is observed in PM [64] and other cancers [65]. Overall, DNA methylation is a powerful approach to identify genes potentially involved in PM and understand its pathogenesis.

Interestingly, both models include CpG sites in noncoding intergenic regions, highlighting the importance of exploring genomic regions beyond protein‐coding genes. These intergenic regions may contain regulatory elements relevant to cancer development, making them valuable biomarkers. This underscores that biomarker discovery is not limited to coding sequences. Moreover, all 22 genes in Model A with multiple CpG sites and all 18 genes in Model B are linked to other cancers, so a single CpG site alone may lack cancer specificity. Combining multiple CpG sites into one model is crucial to increase specificity for PM detection.

Due to the low availability of fresh‐frozen samples, especially for diagnostic confounders, validation experiments were restricted to a limited number of samples. Given the promising two‐step classifier results, broader clinical validation with more tissue samples is essential.

Although the assay itself cannot independently establish a definitive diagnosis of PM, applying this biomarker panel to diagnostic tissue biopsies could enable faster, easier, and more accurate identification of PM when used alongside current methods. Importantly, the current diagnostic gold standard relies on histopathological confirmation of tissue invasion by a pathologist, which remains irreplaceable. Methylation biomarkers cannot directly assess tissue invasion and are therefore not intended to replace this standard. Instead, our panel is designed to complement existing pathological analyses within a multidisciplinary diagnostic framework. By providing molecular insights that may help differentiate PM from other pleural pathologies, particularly in cases where histological interpretation is challenging, it can reduce misdiagnosis and, in turn, inappropriate treatment. Furthermore, the assay's cost‐effectiveness and compatibility with standard laboratory equipment and next‐generation sequencing platforms support its seamless integration into existing diagnostic workflows without requiring specialized or costly infrastructure.

Future efforts will focus on incorporating additional biomarkers to further enhance diagnostic accuracy, particularly in distinguishing PM from other challenging confounders, such as other thoracic malignancies. Additionally, further investigation will evaluate the model's performance on liquid biopsies. These advancements could lead to more precise and earlier diagnosis, ultimately improving patient outcomes and better‐informed treatment strategies.

## 5 Conclusions

In conclusion, we have developed a methylation biomarker assay capable of distinguishing pleural mesothelioma not only from healthy pleura but also from diagnostic confounders such as pleural metastases and chronic pleuritis. Using a two‐step approach, the first model differentiates tumoral from nontumoral conditions with 89.2% sensitivity and 93.5% specificity, and the second model distinguishes pleural mesothelioma from pleural metastases with 85.2% sensitivity and 100% specificity. In clinical settings, this assay shows strong potential as a valuable diagnostic tool to complement current histological analyses on tissue biopsies, ultimately supporting more accurate diagnosis and improved management of pleural mesothelioma.

## Conflict of interest

The authors declare no conflict of interest.

## Author contributions

JV and NDM contributed to conceptualization of the study, experimental lab work, data analysis, interpreting results, writing original draft, reviewing and editing of the final draft. TV and JI contributed to data analysis, reviewing and editing of the final draft. DP contributed to pathological analysis, reviewing and editing of the final draft. SKY, WW, PVS, and JMHH contributed to sample collection, reviewing and editing of the final draft. JR contributed to conceptualization of the study, sample collection, reviewing and editing of the final draft. JM and GVC contributed to conceptualization of the study, interpreting results, reviewing and editing of the final draft. KOB contributed to conceptualization of the study, interpreting results, reviewing and editing of the final draft. All authors have read and approved the published manuscript.

## Supporting information


**Fig. S1.** IMPRESS results of the diagnostic biomarker panel on PM, healthy pleura and healthy blood samples.
**Fig. S2.** Density plot of the cvAUC values of all single smMIP models for the different comparisons.

**Table S1.** Collected samples and their diagnosis.
**Table S2.** Overview of the datasets from which the online available samples are derived.
**Table S3.** Overview of the final smMIP selection and their location in the genome.
**Table S4.** smMIPs of Model A.
**Table S5.** smMIPs of Model B.
**Table S6.** CpG sites of Model A with genomic location and information.
**Table S7.** CpG sites of Model B with genomic location and information.

## Data Availability

Methylation data have been deposited at the European Genome‐phenome. Archive (EGA) and are available on request under accession number EGAS00001008153. Further information about EGA can be found on https://ega‐archive.org. The underlying code for this study is not publicly available but may be made available to qualified researchers upon request from the corresponding author.
