# Supplementary material for: Methylation biomarkers can distinguish pleural mesothelioma from healthy pleura and other pleural pathologies
Source: Mol Oncol. 2025 Nov 14;20(4):933–46. doi: 10.1002/1878-0261.70159 (PMC13060650; doi:10.1002/1878-0261.70159)
Supplement: Supplementary file 1 — Fig. S1. IMPRESS results of the diagnostic biomarker panel on PM, healthy pleura and healthy blood samples. Fig. S2. Density plot of the cvAUC values of all single smMIP models for the different comparisons. [file MOL2-20-933-s001.docx]

**Supplementary Figures**


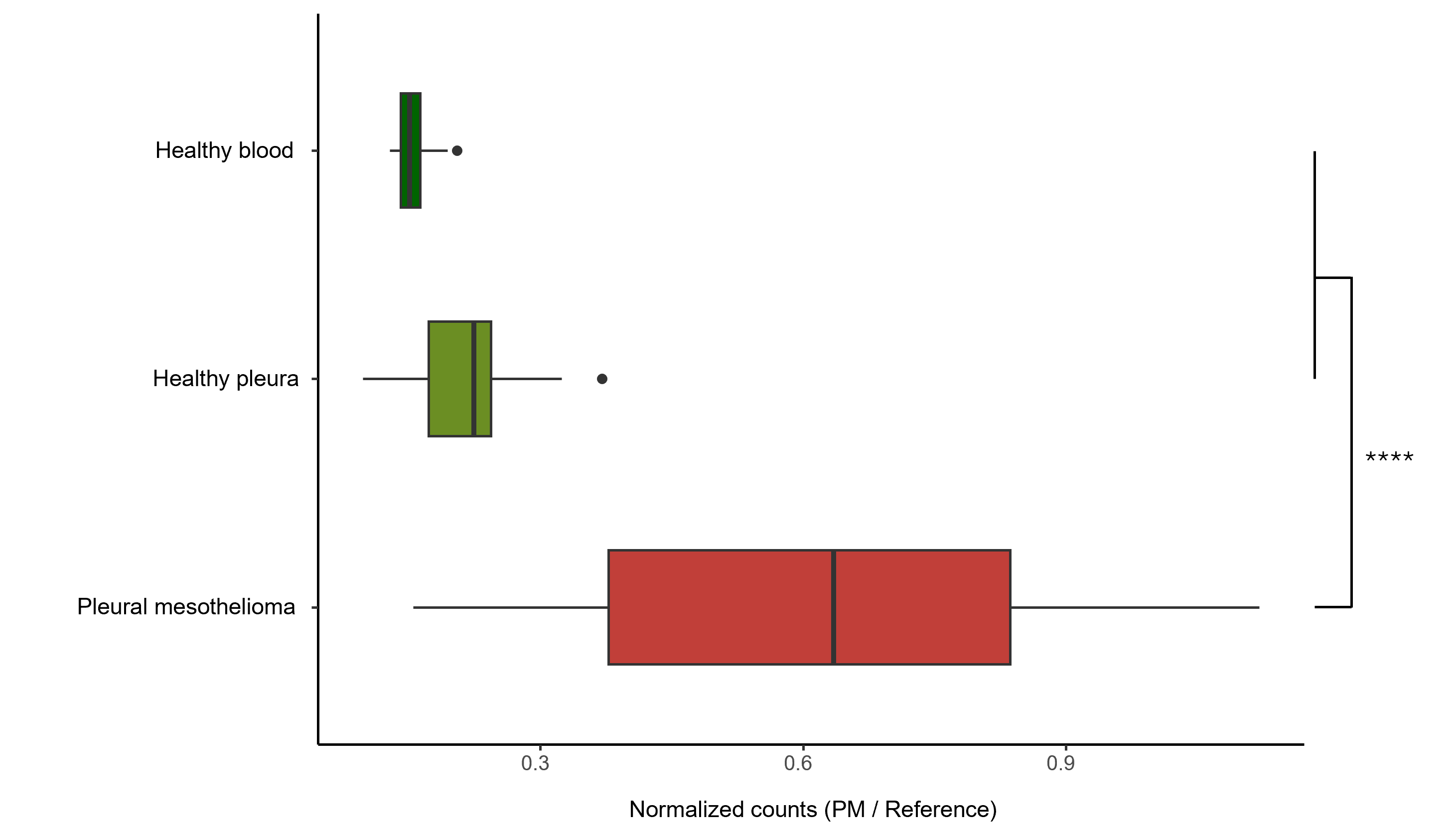


**Supplementary Figure S1 | IMPRESS results of the diagnostic biomarker panel on PM, healthy pleura and healthy blood samples.** The relative methylation level of PM (n=27), healthy pleura (n=23) and healthy blood samples (n=11) are shown. A significant difference in mean normalized counts between PM and healthy samples is shown. **** = *p*-value ≤ 0.0001. The unequal variance (Welch) t-test was performed.


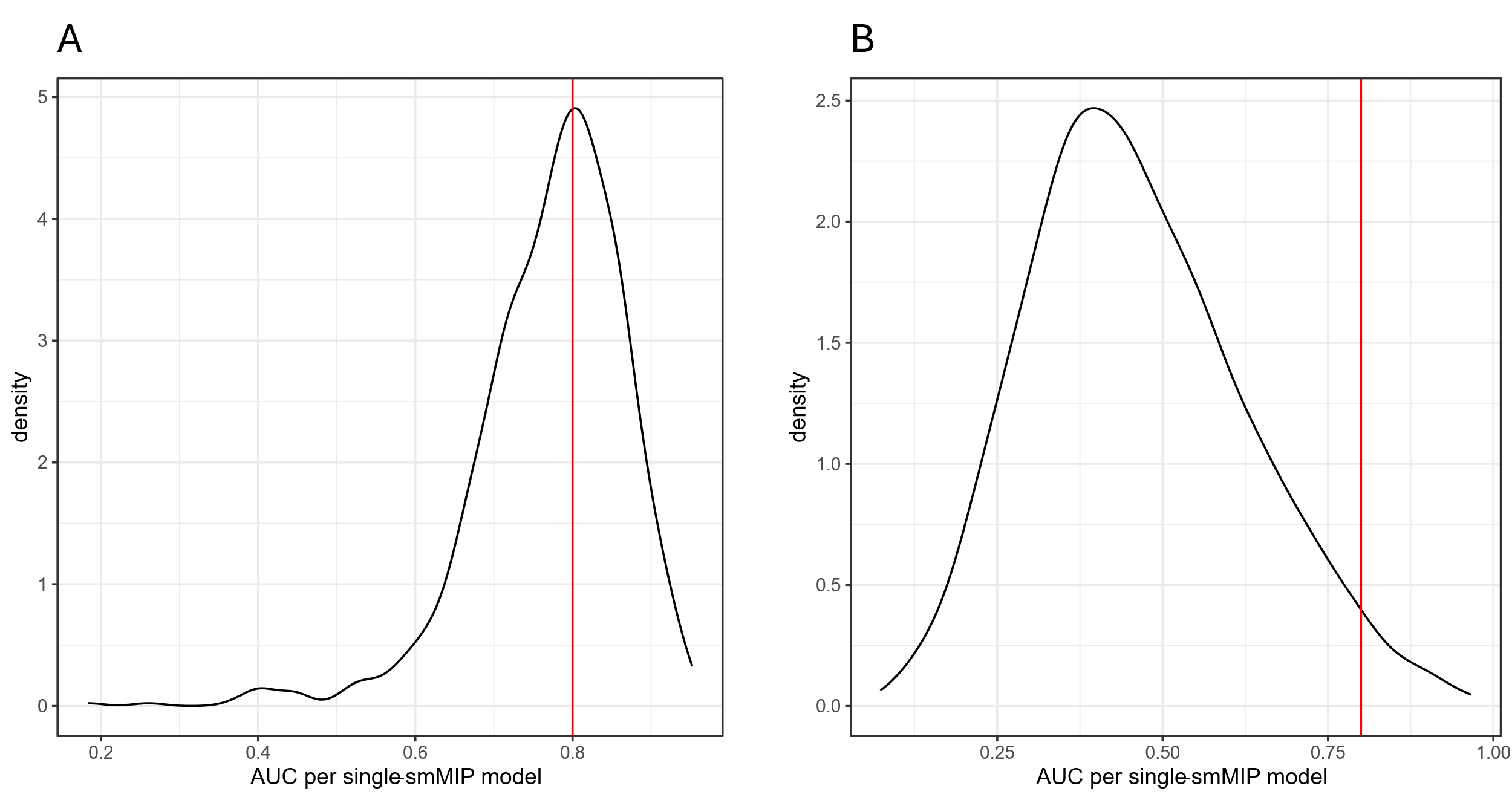


**Supplementary Figure S2 | Density plot of the cvAUC values of all single smMIP models for the different comparisons. (A)** Tumoral (PM and pleural metastases) versus non-tumoral conditions (healthy pleura and chronic pleuritis) and **(B)** PM versus pleural metastases. All single smMIP models with a cvAUC value of at least 0.8 (red cutoff line) were retained for the final model.

**Supplementary Tables**

Supplementary Table S1 | Collected samples and their diagnosis.

Supplementary Table S2 | Overview of the datasets from which the online available samples are derived.

Supplementary Table S3 | Overview of the final smMIP selection and their location in the genome.

Supplementary Table S4 | smMIPs of Model A.

Supplementary Table S5 | smMIPs of Model B.

Supplementary Table S6 | CpG sites of Model A with genomic location and information.

Supplementary Table S7 | CpG sites of Model B with genomic location and information.
